# Neurofeedback and Affect Regulation Circuitry in Depressed and Healthy Adolescents

**DOI:** 10.3390/biology12111399

**Published:** 2023-11-03

**Authors:** Giang H. Nguyen, Sewon Oh, Corey Schneider, Jia Y. Teoh, Maggie Engstrom, Carmen Santana-Gonzalez, David Porter, Karina Quevedo

**Affiliations:** 1 Department of Psychiatry and Behavioral Sciences, University of Minnesota, Minneapolis, MN 55454, USA; nguy2815@gmail.com (G.H.N.); schn1354@umn.edu (C.S.); teohjiayuan325@gmail.com (J.Y.T.); engst263@umn.edu (M.E.); santa079@umn.edu (C.S.-G.); dhp@umn.edu (D.P.); 2 Department of Psychology, Institute for Mind and Brain, University of South Carolina, Columbia, SC 29208, USA; sewon@email.sc.edu

**Keywords:** adolescence, depression, neurofeedback, emotion regulation, amygdala, hippocampus, ACC

## Abstract

**Simple Summary:**

Adolescent depression represents a risk for chronic’ illness when current treatments fail. Given the modest effectiveness of extant therapies, there is a keen need to develop treatments for depression. We used neurofeedback training and positive autobiographical memory retrieval to modulate neural networks that enable emotion regulation and autobiographical memories (amygdala and hippocampus, anterior cingulate cortex) in youth. Our goal was to understand how depressed and control youth engage those regions during emotion regulation and memory recall. Our results showed engagement of the targeted areas as well as differences between diagnostic groups. Future work ought to examine neurofeedback training’s dosage in depressed youth and target cortico-limbic connectivity involved in positive memory recall.

**Abstract:**

Neurodevelopmental psychopathology seeks to understand higher-order emotion regulation circuitry to develop new therapies for adolescents with depression. Depressed (*N* = 34) and healthy youth (*N* = 19) completed neurofeedback (NF) training and exhibited increased bilateral amygdala and hippocampus activity in the region of interest (ROI) analyses by recalling positive autobiographical memories. We tested factors supportive of the engagement of emotion regulation’s neural areas during NF (i.e., parental support, medication, and gender effects upon anterior cingulate cortex (ACC) engagement). Whole-brain analyses yielded effects of NF vs. control condition and effects of diagnosis. Youth showed higher amygdala and hippocampus (AMYHIPPO) activity during the NF vs. control condition, particularly in the left hippocampus. ACC’s activity was also higher during NF vs. control. Higher average ACC activity was linked to better parental support, absent depression, female gender, and absent medication. Control youth showed higher average AMYHIPPO and ACC activity throughout the task and a faster decline in activity vs. depressed youths. Whole-brain level analyses showed higher activity in the frontotemporal network during the NF vs. control conditions, suggesting targeting their connectivity in future neurofeedback trials.

## 1. Introduction

The risk of depression increases throughout adolescence, emphasizing an unmet need to develop effective neuroscience-informed treatments to address depression early [1] and improve long-term outcomes [2,3,4,5,6,7,8,9,10]. Chronic depression that persists post-adolescence is linked to suicide and the economic burden of illness at both individual and health system levels [11,12,13,14]. Depression symptoms correspond with altered brain functions responsible for higher-order tasks. Notably, depressed individuals often exhibit hypo-frontality [15,16], decreased recall of positive memories [17,18], and emotion dysregulation [17,19]. The medial prefrontal cortex aids in the autobiographical recall of emotionally charged memories [20,21]. The anterior cingulate cortex (ACC) facilitates “top–down” emotion regulation [22,23], especially for amygdala-borne emotions [23,24], and it is tightly connected to hippocampus-borne memory functions [25,26]. Yet, the neural underpinnings of these emotion regulation challenges, particularly their potential for non-invasive neuromodulation, are unknown from a neurodevelopmental psychopathology standpoint. 

The present work examines an emotion regulation procedure using a neurofeedback technique targeting limbic and higher-order networks in depressed youth, considering its potential as a future intervention with long-term effectiveness. Real-time fMRI neurofeedback is a procedure where blood-oxygen-level-dependent (BOLD) brain activity and participant visualization occur concurrently with image acquisition. Participants can regulate their hemodynamic activity from regions of interest (ROI) “in vivo”. Therefore, neurofeedback combines endogenous target brain stimulation and emotion regulation strategies supported by areas such as the amygdala, hippocampus, and ACC. Research shows that appropriate cognitive tasks can activate target brain regions [27,28,29,30]. This has shown clinical potential in medicated [31] and un-medicated depressed patients [27,32,33]. Previous research with depressed adults found that a left amygdala-targeted 25 min neurofeedback protocol reduced depression symptoms and engaged frontotemporal cortical areas [27,28,29,33,34].

We sought to adapt the same neurofeedback task [33] for depressed adolescents, considering both neural and developmental factors. We targeted the bilateral amygdala-hippocampus (AMYHIPPO) complex while concurrently recording ACC function. The focus on AMYHIPPO arises from observations that depressed youth display less bilateral mid-temporal limbic activity when recognizing happy self vs. other faces [35]. This region plays a crucial role in adolescent identity formation and affects regulation from a neurodevelopmental psychopathology perspective. Additionally, a hallmark of depression is a blunted experience of positive affect (also known as anhedonia), which is associated with the inability to enhance or recall self-relevant positive experiences [36,37,38,39], potentially due to aberrant hippocampal, amygdala, or ACC functions [40]. Furthermore, AMYHIPPO underlies the retrieval of emotionally charged memories [26,41,42,43,44]. 

Beyond AMYHIPPO dysfunction in depression [45,46,47,48,49], it is essential to consider developmental aspects together. Youth often display increased subcortical limbic engagement like the AMYHIPPO [50], yet regulatory midline cortical structures (MCS), including the ACC, remain immature [51]. The ACC is important for emotion regulation and other higher-order functions [52]. Recalling emotional autobiographical memories activates the ACC [53] and other middle prefrontal areas [54], which are crucial for limbic-driven emotion regulation and depression treatment response [55]. Consequently, we also monitored ACC activity. The immaturity of the ACC and other MCS can result in strong emotions unbridled by regulatory control systems [51]. Moreover, evidence suggests a profound developmental shift from a “bottom-up” to “top-down” fronto-amygdala regulatory connectivity as children progress toward adolescence [23]. Thus, typical adolescent development likely contributes to the onset and severity of depression stemming from a combination of positive affect downregulation, diminished saliency of positive experiences, impaired recall, and immature emotion regulation [50]. 

Neurofeedback research in depressed adolescents is limited. Our group has published the largest sample size to date [52,56,57]. A pilot study [58] of 9 depressed/anxious youths (ages 17–19) showed decreased default mode network (DMN) connectivity after a single mindfulness-based neurofeedback session (1 h 15 min). Another study from Iran [59] with 28 depressed youths taking fluoxetine reported reduced depression after 20 neurofeedback sessions (30 min per session), although the specific procedure remains unclear. To target positive affect regulation via voluntary memory recall grounded in AMYHIPPO and MCS circuits [60,61,62], we shortened a task used by Young et al. [29,33] to reduce participants’ burden and increase engagement. Given the symptom variations between adolescents and adult depression manifestations (e.g., adolescents show fewer vegetative symptoms and less verbalization of hopelessness), consistent brain region engagement is not guaranteed for our study [63]. Therefore, our whole-brain analysis intends to discern potential neural markers for future interventions, investigating if regions responsive in adults similarly engage during adolescent neurofeedback.

Our primary goal was to assess the feasibility of our modified paradigm in adolescents, focusing on the engagement of MCS, especially the ACC, and the reciprocally connected AMYHIPPO. We expected the recruitment of a similar frontotemporal cortical network, as Young et al. [29,33] reported, supporting interoceptive awareness, self-processing, emotion regulation, and memory retrieval. Given the crucial role the AMYHIPPO plays in encoding positive memories and detecting emotional intensity [26,64,65] and its connection with MCS like the medial prefrontal cortex and ACC [41,42,44,65,66,67], we anticipated that depressed youth would show reduced engagement in these areas, reflecting deficits typical in depression [46,68], such as impaired executive function, positive emotion regulation, and positive self-referential memories [69,70]. 

Another goal was to understand the association between emotion regulation circuitry in youth during neurofeedback, particularly ACC activity [53], and parent-reported co-regulation measures. Established research indicates that nurturing parental relationships and adequate co-regulation of emotions foster teenagers’ affect regulation [71,72]. Early co-regulation experiences leave lifetime effects [71], with maladaptive emotion regulation in children mediating the relationship between negative parental conflict resolution styles and their internalizing and externalizing problems [73]. Therefore, we posited that parental support for children’s emotions would correlate with adolescents’ emotion regulation circuitry (ACC, AMYHIPPO) during neurofeedback in ROI analyses.

Our hypotheses were: (1) All youth would exhibit higher AMYHIPPO and ACC activity during neurofeedback vs. count-backward in ROI analyses using hierarchical linear modeling; (2) Activity in frontotemporal cortical areas would be higher during neurofeedback vs. count-backward; (3) Higher parental emotional support would predict higher engagement of emotion regulatory networks (e.g., ACC); (4) depressed adolescents would differ from controls in AMYHIPPO or ACC activity during neurofeedback, possibly by showing less activity [46,68]. Given the lack of prior NF research with adolescents, we had no strong directional or anatomical hypothesis for differences in ROI (AMYHIPPO, ACC) or whole-brain activity between depressed and control groups. 

## 2. Materials and Methods

This study was conducted at the University of Minnesota (U of M) Center for Magnetic Resonance Research with approval from the U of M Institutional Review Board. Fifty-three right-handed neurofeedback-naive adolescents with (*N* = 34, *M*_age_ = 16.11) or without (*N* = 19, *M*_age_ = 16.35) depression were enrolled and evaluated in both categorical (Kiddie Schedule for Affective Disorders and Schizophrenia—Present and Lifetime Version; K-SADS-PL) [74] and continuous (Children’s Depression Rating Scale; CDRS) [75] measures of the presence of mood disorder and depression severity during the first intake session (S1). Table 1 contains the demographics and clinical backgrounds of the participants. We did not incorporate a placebo group, as preliminary studies do not necessitate it [76]. Parents’ reports on support provided to their child (when the child was experiencing sadness, anger, and fear) were obtained via the Emotional Socialization Measure (ESM) [77]. Additional details of inclusion and measures are in Supplementary Materials S9. During the second session (S2), experimenters guided participants to select and write 5–6 positive memories and identify the peak 10–15 min of the highest positive mood during each event. These memories were revisited prior to the start of the “Happy” word neurofeedback training. This task was presented in a counterbalanced manner [52] alongside another neurofeedback task on self-versus other faces. Participants were encouraged to use either the same or different memories across both tasks to achieve maximum target engagement. A comprehensive description of these additional tasks can be found in our previous publications [52,56,57]. 

### 2.1. “Happy” Word Neurofeedback Task 

**Figure 1:**
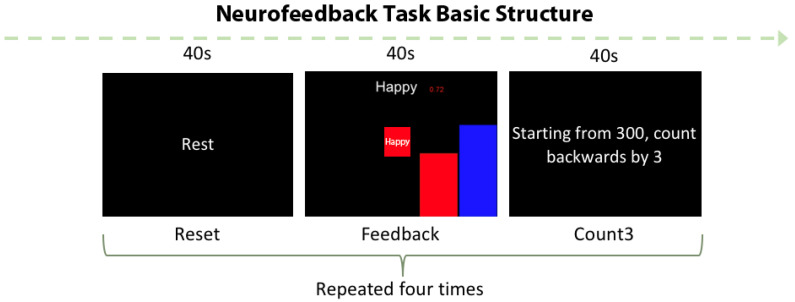
“Happy” word neurofeedback task. Participants recalled happy memories to the cue of the word “happy” and counted backward from 300 by 3 during the control condition. The blue bar is a guide for the changing activity displayed via the red bar.

This task is a shorter adaptation (520 s) of a ~25 min protocol tested in adults by Young et al. [29,33]. Our task was comprised of rest, neurofeedback (NF), and count-backward (CB) conditions that were presented for 40 s each per block. They appeared sequentially for four blocks (Figure 1). The task was delivered via PsychoPy2 software [78]. During the NF condition, participants saw the word “happy” and attempted to increase AMYHIPPO activity displayed by a red bar shifting up or down depending on values provided using MURFI software [79]. MURFI updated and displayed values at the same time of each brain volume acquisition, and the bar was updated accordingly. A static blue bar to the right offered a guide for desired levels of AMYHIPPO activity. To increase AMYHIPPO activity, participants recalled happy memories discussed prior to the scanning. Participants were notified about the 2-s delay between BOLD-measured effortful imagination and visible effects displayed by the red bar. They were also instructed to change the positive memory if the one they were recalling was not effective. Participants were not told how long to persist with a single memory, just to change it if it seemed not to be effective. During the CB condition, participants were instructed to count numbers backward by three starting from 300 with no NF.

The count-backward condition in our task served as a control condition, targeting working memory areas [80] without invoking emotional or self-referential processes. Both active (i.e., count-backward) and non-active (i.e., rest) conditions are used as contrasts in ROI and whole-brain level analyses during neurofeedback protocols.

### 2.2. Online Analysis: Real-Time Feedback from the Brain

MURFI software [79] generated and sent estimated ROI activity values to the scanner screen during the feedback conditions following every TR (2100 ms.) when each new volume was acquired. The neurofeedback signal was provided from a combination of AMYHIPPO ROI and subject-specific anatomic masks (Supplementary Figure S1 for an example) of the bilateral AMYHIPPO generated with the WFU PickAtlas 3.0 software tool [81]. This mask was transformed from the MNI space into the subject-specific functional imaging space via a 4-step process using SPM12 modules [82]. This four-step process is detailed in Supplementary Materials S1. Using this process, experimenters observed real-time fluctuations in both AMYHIPPO and ACC activity, while participants only saw feedback from AMYHIPPO activity. MURFI estimates and generates neural and nuisance signals using incremental linear least squares GLM fit, which estimates the raw signal in each voxel at each time point and scales corrected by the noise [79]. So, neurofeedback was continuously updated with each TR. The main ROI analysis of AMYHIPPO and ACC activities was conducted using the mean signal for each of the conditions with volume of interest extractions for the whole time series using the subject-specific ROIs after pre-processing and correction for movements of >2 mm, which is an acceptable standard for pediatric clinical populations. 

### 2.3. Off-Line Analysis: Whole Brain Analysis

Pre-processing and first-level analyses are described in Supplementary Materials S2. A voxel-wise model with condition (NF vs. CB), diagnosis (depressed vs. controls), and IQ as additional predictors was used to identify neurofeedback effects using a family-wise error (FWE) corrected cluster level threshold at *p* < 0.01. Voxel-wise analysis of the neurofeedback training period was performed using the first-level NF vs. CB contrast in a single-sample *t*-test with FWE-corrected peak-level threshold at *p* < 0.01. 

A full factorial general linear model (GLM) with neurofeedback tasks as within-subjects conditions (NF, CB) was used to identify group and group by conditions effects. Participant’s IQ was inserted as a covariate that differed between the groups (Table 1). A combined voxel-height and cluster-extent threshold was calculated to control for Type 1 error using Monte Carlo simulations in Analysis of Functional NeuroImages (AFNI v.18.2.06) [83]. Using 3dClustSim with α = 0.01 and *p* < 0.001 for the principal GLM, only clusters with a minimum of 152 voxels per cluster were significant. Smoothness estimates entered in 3dClustSim (12.28 11.38 13.39) were calculated using linux program 3dFWHMx.

Additional analyses were conducted comparing NF and baseline (i.e., rest) conditions to verify the effects of the NF condition as significantly higher than rest times and are reported in Supplementary Materials S3 and Figure S2. 

### 2.4. Off-Line Analysis: AMYHIPPO and ACC Activity ROIs Analyses

We anticipated both AMYHIPPO and ACC increased activity during neurofeedback. To test our hypothesis, ROI analyses were conducted using both linear mix models in SPSS 25 and image *t*-tests analyses in SPM12. 

Each subject’s first-level activity maps and subject-specific AMYHIPPO or ACC masks were used to derive a mean within-subject BOLD activity value for the NF and CB conditions. The value was incorporated to test variables that were associated with activity during the “Happy” word task. Linear mixed models (LMM) were used to analyze AMYHIPPO and ACC activity during the neurofeedback task using SPSS 25 (Supplementary Materials S4). LMM was used to estimate individual intercepts as random effects, mean intercepts, and slopes (e.g., linear or quadratic slope) as fixed effects and parameters for predictors of interest (i.e., diagnosis, parental support, gender, medication presence). 

The linear decrease over time (which coexisted with significant modulation for neurofeedback vs. count-backward) and inflection points observed in the average time series corresponding to those activity increase and decrease (Supplementary Materials S4) were modeled with a linear, quadratic, or cubic parameter as needed (Equations (1) and (2)). 

Equation (1) (Level 1):

(1) $${AMYHIPP\;or\;ACC}_{i}=\beta 0_{i}+\beta x_{i}+\delta$$

Equation (2) (Level 2):

(2) $$\beta 0_{it}=\gamma 00+\delta 0_{i}\;\beta 1_{it}=\gamma 01+\delta 1_{i}\;\beta 2_{it}=\gamma 02+\delta 2_{i}\;\beta 3_{it}=\gamma 03+\delta 3_{i}\;\beta x_{it}=\gamma 0x+\delta x_{i}$$

where δ = error, t = time point, i = subject, linear (γ01) = 0, 1 to 8, quadratic (γ02) = 0, 1, 4 to 64, cubic (γ03) = 0, 1, 8, to 512. x = predictors of interest (e.g., Diagnostic Group, Gender, Medication, IQ, Parental Support, etc.).

Data visualization and covariance tests suggested that an identity structure for the random effects covariance structure was the best fit for modeling. LMM was tested using 17 initial predictors. Departing from that model, variables were removed one at a time, starting with the least significant. The resulting nested smaller models were compared to the prior larger ones via a χ^2^ goodness of fit test using the −2 log-likelihood difference. Type 3 F tests of fixed effects are reported in Supplementary Materials S5 and Table S2. Significant parameter estimates are reported in Table 2. All final predictors in our results converged toward a model for AMYHIPPO or ACC activity. Only significant predictors are displayed, which also converged toward the best goodness of fit final model tests. For example, medication was a significant predictor of ACC activity but not of AMYHIPPO. It was included as an AMYHIPPO predictor in the initial model but was removed due to lack of significance. The goodness of fit tests generates the simplest model with the least predictor’s number that significantly accounts for a dependent variable variance.

For image activation analyses, contrasts of neurofeedback versus count-backward and neurofeedback versus baseline (i.e., rest) were created for each participant to confirm whether AMYHIPPO activation was significant. One-sample t-tests (including diagnosis, IQ, and gender as covariates) were conducted in SPM12 on the group level with cluster forming threshold of *p_uncorr_* < 0.001 and a small-volume corrected threshold of *p*_FWE_ < 0.05 for the AMYHIPPO ROI. 

## 3. Results

### 3.1. “Happy” Word Neurofeedback Task: AMYHIPPO ROI Analysis 

**Figure 2:**
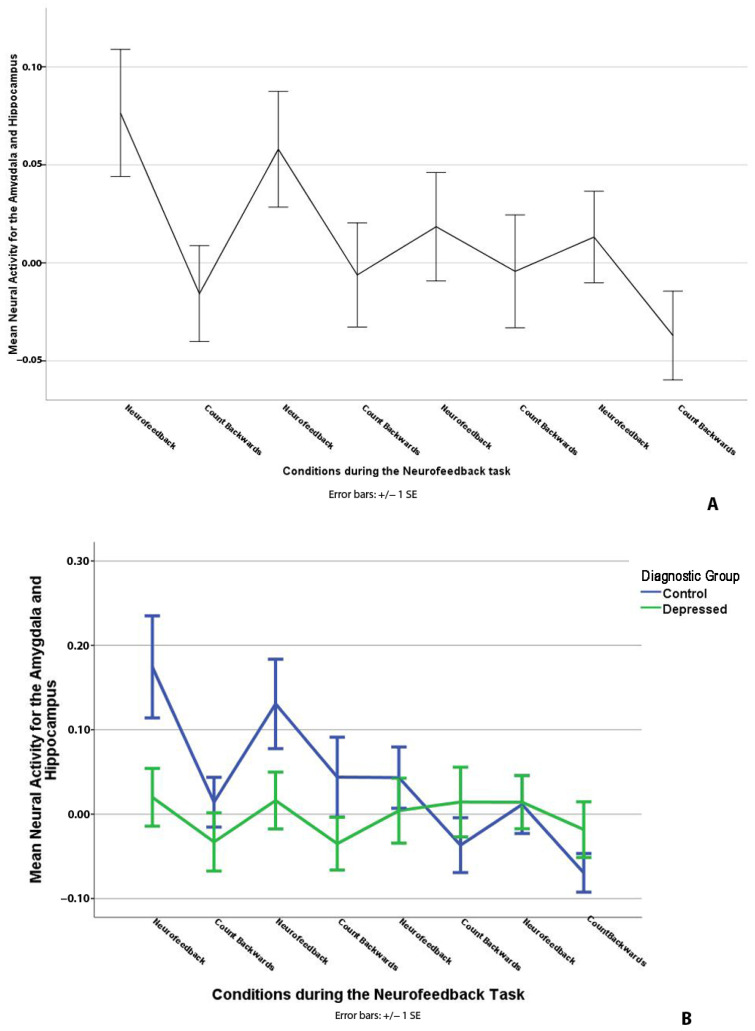
(**A**) Main effect of the condition confirmed a successful neurofeedback training effect as evidenced by higher AMYHIPPO during neurofeedback vs. count-backward condition. (**B**) Control youth shows higher average AMYHIPPO activity during the “Happy” word neurofeedback task, and stepper activity decreases over time compared to depressed youth. SE = standard error of the mean.

A significant effect of task condition on mean AMYHIPPO activity was observed, *t*(364) = 2.96, *p* < 0.01, demonstrating a higher AMYHIPPO activity during the neurofeedback compared to the count-backward condition during the “Happy” word task (Table 2, Figure 2A). Healthy adolescents (Control) exhibited higher average AMYHIPPO activity compared to depressed youth during the entire time series, *t*(203.96) = 3.50, *p* < 0.01 (Table 2, Figure 2B).

Controls’ AMYHIPPO activity decreased over time throughout the task, *t*(364) = −4.02, *p* < 0.01, whereas depressed youth showed no notable change, *t*(364) = 0.52, *p* = 0.60 in AMYHIPPO activity over time (Table 2, Figure 2B). Planned contrasts confirmed this finding (Controls vs. Depressed slope difference) = −0.242, *p* < 0.001. Of note, a diagnosis by AMYHIPPO time series slope interaction is present in addition to the main effect of task condition (i.e., higher ROI activity for neurofeedback vs. count-backward). It does not mean failed neurofeedback training. It means different average activity changes for the diagnostic groups over time in addition to an NF task condition effect (i.e., higher average activity during NF vs. CB and a decline in activity over time, as noted inFigure 2A).

**Figure 3:**
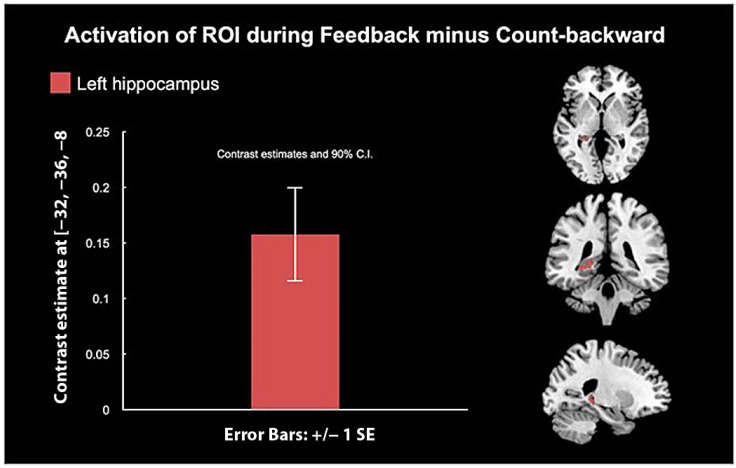
AMYHIPPO ROI analysis in SPM 12 for neurofeedback vs. count-backwards showed a positive activity difference in the left hippocampus. Suggesting that this area was significantly modulated during the NF condition. SE = standard error of the mean.

The small volume corrected ROI analysis for NF vs. CB conditions in SPM12 revealed a positive difference in the left hippocampus activity (Figure 3). ROI analysis of NF versus rest yielded no significant results. 

### 3.2. “Happy” Word Neurofeedback Task: ACC ROI Analysis

**Figure 4:**
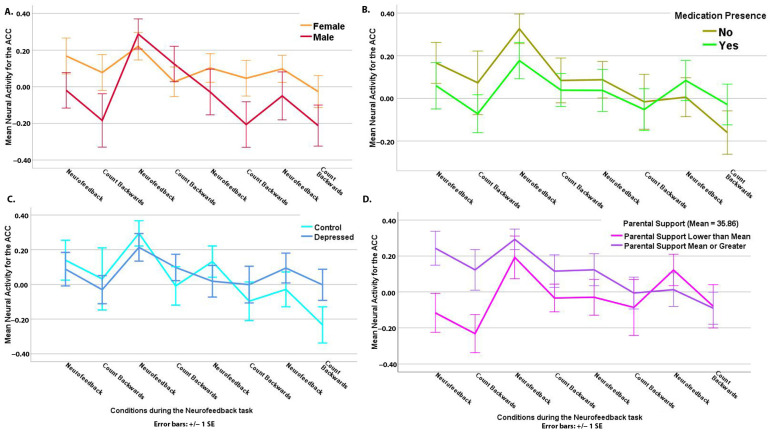
(**A**) Females show higher average ACC activity vs. males. (**B**) Medication was associated with lower average ACC activity. (**C**) Controls show stepper negative decreased ACC activity over time versus depressed. (**D**) Higher parental emotional co-regulation was associated with higher average ACC activity. SE = standard error of the mean.

Table 2 shows that, as with the AMYHIPPO target, all youth had higher ACC activity during the neurofeedback vs. the count-backward condition, *t*(349.24) = 2.45, *p* < 0.05 (Table 2, Supplementary Figure S3). There was a main effect of gender, *t*(50.23) = 2.38, *p* < 0.05. The average ACC activity was higher for females compared to males, *t*(50.23) = 2.38, *p* < 0.05 (Table 2, Figure 4A), and un-medicated youth had higher overall ACC activity compared to medicated youth, *t*(102.06) = 2.40, *p* < 0.05 (Table 2, Figure 4B).

A diagnosis by linear slope interaction showed (again similar to AMYHIPPO) a steeper decline in ACC activity during the overall task time series for control, *t*(393.87) = −3.50, *p* < 0.01, in contrast to depressed youth, *t*(395.90) = −0.07, *p* = 0.94 (Table 2, Figure 4C). Planned contrasts confirmed this finding (Controls vs. Depressed slope difference) = −0.05, *p* < 0.001. 

As hypothesized, higher parental support (i.e., better co-regulation of a youth’s negative emotions by the guardian’s self-report) was associated with higher average ACC engagement during the entire task time series, *t*(49.71) = 2.69, *p* < 0.01 (Table 2, Figure 4D).

### 3.3. “Happy” Word Neurofeedback Task: Whole-Brain Analysis

**Figure 5:**
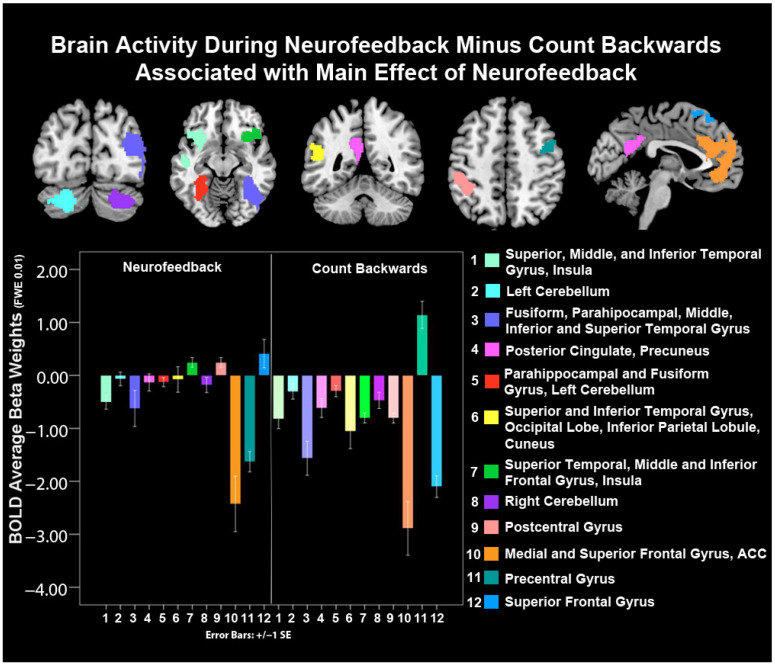
There is less average de-activation during NF vs. CB conditions in frontotemporal and limbic structures. Note: Follow-up *t*-tests (Supplementary Materials S6 and Table S3) showed higher precentral gyrus (BA 11) during NF vs. CB (coordinates: 38 −04 46), despite the appearance of more activity in this figure for CB). SE = standard error of the mean.

An effect of neurofeedback (NF) vs. count-backward (CB) conditions in GLM analyses showed that the right superior temporal, middle, and inferior frontal gyrus, and insula, as well as the left post-central gyrus and superior frontal gyrus, BA 6, 9 show significantly higher average activity during NF vs. CB conditions. These are bolded in Table 3 and depicted in Figure 5: green (7), light pink (9), and blue (12) bars, respectively.

On average, youth showed less de-activation during NF versus CB (i.e., higher relative average activity) in the left superior, middle and inferior temporal gyrus, insula, cerebellum, fusiform, parahippocampal, middle inferior and superior temporal gyrus, left posterior cingulate and precuneus, occipital lobe, inferior parietal lobule, cuneus, right inferior frontal gyrus, insula, and the ACC including the dorsal and ventral aspect of this key regulatory structure, except for precentral gyrus (Figure 5, Table 3).

**Figure 6:**
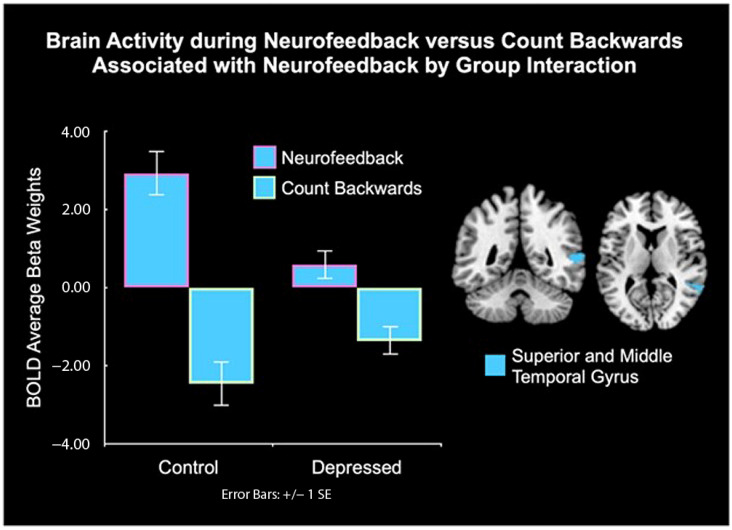
Controls showed higher activation in the superior and middle temporal gyrus during NF vs. CB compared to depressed youth. SE = standard error of the mean.

A group-by-condition interaction showed that controls had higher activity during NF vs. CB in the right superior and middle temporal gyrus (SMTG) compared to depressed youth (Figure 6, Table 3). Follow-up t-tests confirmed this direction and interaction (Supplementary Materials S6 and Table S3). 

Whole brain GLM analyses regarding activity during NF vs. Rest are reported in Supplementary Materials S3 and Figure S2. Additional *t*-tests are unnecessary, as the rest of the conditions are an implicit baseline for our objectives and stated hypothesis.

## 4. Discussion

### 4.1. Amygdala and Hippocampus (AMYHIPPO) and Anterior Cingulate Cortex (ACC) Activity during Neurofeedback

Our research study used a region of interest (ROI) analysis methodology similar to previous neurofeedback studies to examine changes in neural activity during neurofeedback training. For example, a neurofeedback study with depressed adults used ROI analysis to examine amygdala connectivity after neurofeedback [84]. A recent neurofeedback study on depressed patients also used ROI analysis after cognitive-reappraisal neurofeedback training [85]. These and other studies [86,87,88] show the prevalence of ROI analysis methodology to demonstrate neural engagement specificity in areas of interest during, before, and after neurofeedback. 

Adolescents showed higher AMYHIPPO and ACC activity during neurofeedback (NF) vs. count-backward (CB) conditions in ROI analyses, confirming target engagement (Figure 2A) during neurofeedback. Small volume corrected ROI analysis showed higher left hippocampus activity during the NF vs. CB conditions and no significant activity for the right hippocampus or the amygdalae. Higher left hippocampus engagement may underpin voluntary recall of positive autobiographical memories [89,90] during neurofeedback in youth. Along with the ACC, the left hippocampus might be easier to train during adolescent’s neurofeedback training, entailing memory recall and the “Happy” word task. Higher AMYHIPPO activity during NF vs. CB on average for this adolescent sample (Figure 2A) during LMM is consistent with prior adult neurofeedback studies that employed recall of happy autobiographical memories [34,91,92,93] and with most neurofeedback research that employs ROI analysis. 

Controls showed higher mean AMYHIPPO and ACC activity in ROI analysis during the neurofeedback time series, as well as a steeper decline over time compared to depressed adolescents. Depressed individuals are less adaptive in selecting and using emotion regulation strategies than healthy ones [94,95] and have known impairments in recalling and elaborating positive memories [18,38,96]. Perhaps control youth regulated their emotions more flexibly than depressed youth and/or are better able to engage “top-down” regulatory structures to influence AMYHIPPO activity during neurofeedback [67]. Positive memory recall difficulties are linked to depression [18,96]. This likely influenced less flexible cortical-limbic ROI engagement among depressed youth during neurofeedback. Of note, there were no group differences reported on ease of memory recall (Supplementary Materials S7). However, differences in memory richness and elaboration [37] and ability to elicit accompanying positive emotions [17,39] likely remained. 

A group by linear slope interaction showed that AMYHIPPO and ACC activity decreased over time for the control youth, but neither was associated with time for the depressed youth (Figure 2B and Figure 4C). Decreasing activity over time suggests a practice and/or habituation effect, common for limbic regions [97] that underpin arousal and saliency [98,99]. If this is the explanation, this is consistent with a more flexible engagement of cortical-limbic networks among control vs. depressed youth during the neurofeedback task time course [94]. Thus, decreasing overall activity over time is not to be interpreted as “failed neurofeedback” first because this is a time-series analysis of one single session. Targeted regions’ engagement has been reported for more than one neurofeedback training session in past adult research [27,33] that did not examine single-session time-series engagement for the target ROI [27,33]. Second, the decreasing activity over time occurred alongside significantly higher ROI engagement for neurofeedback vs. count-backward conditions, indicating successful differential brain modulation as intended by the procedure.

ACC activity was also higher during the neurofeedback versus count-backward condition in ROI analyses (Supplementary Figure S3). However, medication absence was associated with higher average ACC activity during neurofeedback. Medication might be a proxy for diagnosis, but its absence is associated with increased ACC engagement at all other predictors’ average levels (as interpreted using LMM). While our modeling identified ACC activity predictors over the time series, covariances between medication and diagnosis must be examined with larger samples to test un-medicated vs. medicated depressed youth’s neurofeedback performance. Medication in this sample co-varied with depression. Specifically, most depressed participants were medicated, while control youth were not (Table 1). However, it must be noted that when diagnosis was used as an ACC activity predictor instead of medication, the association between diagnosis and ACC activity was not significant, and the resulting model did not fit the data. Removing medication resulted in a worse model fit to ACC time-series data during the neurofeedback task. Information regarding medication types is provided in the supplements (see Supplementary Materials S8 and Table S4). Future research should compare depressed non-medicated and medicated youth to tease out the interaction of medication and neurofeedback upon neocortical regulatory engagement, e.g., ACC, in clinical pediatric samples.

Females showed higher average ACC activity compared to males. Effortful conscious cognitive regulation depends on ACC engagement, which allows for the modulation of limbic systems [100]. Therefore, girls might have exerted more cognitive effort [101], experienced more conflict [102], monitored their experiences, or engaged in more successful emotion regulation [103] during the neurofeedback task. Future research ought to include a larger number of depressed and control boys to test this explanation regarding gender differences.

As predicted, parental support (i.e., better co-regulation of offspring’s negative emotions) was associated with higher average ACC activity. However, parental self-reported co-regulation was unrelated to AMYHIPPO activity. Given the positive effects of parental support on offspring emotion regulation [71,72], higher overall ACC activity indicates better emotion regulation during voluntary emotion regulation via neurofeedback. Thus, combining interpersonal therapy and/or parent–child emotion co-regulation guidance with neurofeedback or on its own will positively influence brain function among depressed adolescents.

### 4.2. Whole-Brain Analysis

The right frontal gyrus and insula and left post-central and superior frontal gyri, BA 6, 9 showed significantly higher average activity during NF vs. CB conditions, consistent with neurofeedback studies showing increased pre and postcentral gyri activity when children attempted to voluntarily regulate brain function [104]. Positive memory recall using a higher dosage of neurofeedback was linked to increased amygdala, hippocampus, cuneus, insula, and ACC activity [92,93] during the “Happy” word task. 

Ours and prior work suggest that during neurofeedback, children and adolescents engage in areas that support emotion regulation and self-processing [i.e., midline cortical structures such as the superior frontal gyrus, BA 9 [70,105,106], memory retrieval [40], interoceptive awareness insula [107], and behavioral preparedness and/or representation of actions postcentral gyri [108]]. Recalling positive memories might engage action representations associated with such events. The insula and ACC are critical components of the salience network, supporting social behavior and self-awareness [109]. Increased right insula activation during neurofeedback warrants research on this and other tasks in depressed youth. Specifically, increasing hippocampus to superior frontal gyrus and insula connectivity via neurofeedback training. For this task, the insula, post-central gyrus, and superior frontal cortex connectivity with the left hippocampus might be good neurofeedback targets in adolescents. 

There was less de-activation in multiple cortical areas during NF vs. CB. Multiple explanatory models have been advanced to account for BOLD de-activation, a frequent finding in fMRI research [110]. Further investigation with longer task durations and higher neurofeedback dosages may be needed to elicit higher activity in adolescents. Alternatively, longer baseline (rest) periods may be needed to properly elicit activity contrasts during NF and CB that differ from rest conditions as an implicit baseline. 

### 4.3. Diagnosis and Brain Activity during Neurofeedback

Controls showed higher superior and middle temporal gyrus (SMTG) activity during the NF vs. CB condition compared to depressed youth. To rule out whether this was due to IQ differences between the diagnostic groups, we tested the effects of IQ. Results showed that the effects of IQ did not overlap with areas differing between the groups in whole brain activity during the task. The SMTG is involved in social cognition functions such as mentalization and agency inference [41,111,112], suggesting that controls might engage in higher mentalization while recalling positive autobiographical memories during NF during this task compared to depressed youth. 

To our knowledge, this is one of the first neurofeedback studies of depressed adolescents targeting the neural basis of positive autobiographical memories. Unlike adult results, we found no whole-brain level activation of the bilateral amygdala and hippocampus. However, we did observe higher activity in those ROIs during NF versus CB conditions and engagement of midline cortical structures such as the superior frontal gyrus and insula. Our results are useful for future neurofeedback trials in youth targeting those areas with a shorter neurofeedback paradigm. 

## 5. Limitations and Future Studies

Unlike depressed adults’ [27,29,33] whole-brain level engagement of the left amygdala, this adolescent sample did not show increased AMYHIPPO activity in whole-brain level analysis during an emotion regulation neurofeedback “Happy” word task for either NF compared to CB or baseline. One explanation is the shorter training duration (5 min) compared to 25 min of neurofeedback training undergone by adults [33], i.e., an issue of dosage. Alternatively, adolescents, unlike adults, might be unable to modulate these areas voluntarily to an extent visible in whole-brain level analyses. Thus, different areas (e.g., dorsal ACC, superior frontal cortex, or insula) ought to be targeted to observe whole-brain level results, especially because these limbic areas are relatively smaller and more prone to be affected due to their deep positioning in the brain. However, a shorter duration for a preliminary study was a developmentally sensitive adjustment to facilitate youth’s participation while performing a mental task with low visually rewarding stimulation. An additional source of differences from adult results may stem from the “Happy” word task design not being rewarding enough to engage adolescents. A neurofeedback task completed by this sample (in counterbalanced order), which used faces (self or others) to cue NF or CB, respectively, elicited stronger results [52], hinting at the potential effects of more stimulating designs for youth. A combination of these factors likely explains the absent whole-brain level activity in the targeted limbic areas. From a scientific standpoint, both dosage and the rewarding/stimulating quality of neurofeedback ought to be researched to refine this procedure’s use in pediatric clinical samples. Finally, another critical explanation is the known functional differences between children, adolescents, and adults during emotion regulation [50,113]. A recent study found that higher ACC activation increases amygdala reactivity in childhood but decreases in adolescence, along with the nature of the information flow from the amygdala to PFC and ACC in childhood that flips for adolescence [23]. Thus, explicit emotion regulation might be engaging higher function neural areas (e.g., superior frontal gyrus, BA 9) to a greater extent than the targeted limbic ones in youth, as is apparent in whole-brain analyses.

This study lacked a placebo group. Our goal was to test neurofeedback in a pediatric sample while comparing their activation and performance to healthy adolescents in key brain regions. Therefore, we cannot rule out the possibility that changes in amygdala and hippocampus complex and/or frontotemporal activity are associated with the mental activity itself (i.e., positive memory recall). A placebo group, i.e., neurofeedback provided from an area unrelated to the task, would ascertain whether neurofeedback in adolescents is associated with symptom improvements. Another limitation is the absence of baseline and transfer runs (i.e., pure mental imagery without neurofeedback). Given that this was an adaptation of a longer neurofeedback task done for adults and that our goal was to test feasibility, neural function during neurofeedback in adolescents, and differences in emotion regulation between controls and depressed youth, we shortened the task to fit our goals and tested in counterbalanced order another neurofeedback task. Therefore, we cannot provide any definite conclusion regarding emotion regulation learning. The small sample size is another limitation of this study and should be considered when interpreting the results. Future studies of adolescent emotion regulation with neurofeedback would benefit from a larger sample size and the inclusion of transfer and baseline runs as well as a placebo group.

## 6. Conclusions

Neurofeedback targeting the AMYHIPPO via recall of positive autobiographical memories engaged mid-cortical areas that support emotion regulation and self-processing (i.e., insula, pre-motor, and superior frontal cortex). These areas enable interoception, memory retrieval, and motor function in both depressed and healthy control adolescents. Healthy controls might more flexibly engage cortical and limbic targeted areas during neurofeedback. They may be more able to modulate amygdala and hippocampus (and possibly ACC) activity than depressed adolescents. Our results suggest that at least two longer-lasting sessions of amygdala and hippocampus or just left hippocampus neurofeedback procedure should be tested in the future among depressed youth.

## Figures and Tables

**Table 1:** Demographics and statistics of participants.

	Controls	Depressed	Statistics
	*N* = 19	*N* = 34	
**AgeS1**: M (SD)	16.26 (1.19)	16.08 (1.27)	*F*(1, 48) = 0.26
**AgeS2**: M (SD)	16.35 (1.23)	16.11 (1.25)	*F*(1, 48) = 0.45
**IQ**: M (SD)	115.32 (9.12)	108.35 (10.84)	*F*(1, 51) = 5.61 *
**Sex**			χ^2^(1) = 0.31
Male	7 (36.84%)	10 (29.41%)	
Female	12 (63.16%)	24 (70.59%)	
**Puberty**: median (IQR)	4.75 (0.50)	5.00 (0.50)	U(19, 33) = 276.5
**Ethnicity**			χ^2^(4) = 7.69
White	14 (73.68%)	27 (79.41%)	
African American/Black	0	2 (5.88%)	
American Indian	0	2 (5.88%)	
Asian	3 (15.79%)	0	
Other	2 (10.53%)	3 (8.82%)	
**Family Structure**			χ^2^(3) = 2.80
Married	15 (78.95%)	22 (64.71%)	
Living with partner	1 (5.26%)	3 (8.82%)	
Separated-Divorced	3 (15.79%)	5 (14.71%)	
Single	0	4 (11.76%)	
**Income**			χ^2^(2) = 3.90
=>35 K	0	6 (17.65%)	
35–75 K	7 (36.84%)	9 (26.47%)	
+>75 K	12 (63.16%)	19 (55.88%)	
**Medication**			
Antidepressants	0	26	
Antipsychotics	0	2	
Mood stabilizers	0	0	
Anxiolytic	0	10	
**Depression Severity**: *M* (*SD*)/median (IQR)	19.21 (3.56)/18 (2.5)	49.85 (16.14)/48.5 (28.5)	*F*(1, 51) = 66.06 **
**Parental Support**: *M* (*SD*)	37.95 (5.02)	34.82 (7.14)	*F*(1, 50) = 1.45

* *p* < 0.05, ** *p* < 0.01; S1: intake screening session; S2: scanning neurofeedback session; *M* = mean; *SD* = standard deviation; the data that does not follow normality are reported as median and IQR = interquartile range. *F*: F test statistics; χ^2^ = chi-square test statistics; U = Mann–Whitney U test (also known as Wilcoxon Rank Sum test) statistics; Depression Severity was measured with Children’s Depression Rating Scale; Parental support was measured with Emotion Socialization Measure; median and IQR combination is reported when the data did not follow normality; Data on depression severity did not follow the normality only for the control group, so both combinations of the descriptive statistics are reported.

**Table 2:** Parameter estimates from linear mixed models predicting AMYHIPPO and ACC activity during neurofeedback task.

Predictors of Mean AMYHIPPO Activity during the Neurofeedback Task’s Time Series					
Effect	Estimate *γ*	SE *γ*	*df*	*t*	*p*
Intercept	0.01	0.03	212.53	0.43	0.67
Group					
Controls	0.16	0.05	203.96	3.50	<0.01
Depressed					
Condition					
Count Backwards					
Neurofeedback	0.05	0.02	364	2.96	<0.01
Group by Linear Slope					
Controls × Linear Slope	−0.24	0.01	364	−4.02	<0.01
Depressed × Linear Slope	0.002	0.005	364	0.52	0.60
**Predictors of Mean ACC Activity during the Neurofeedback Task’s Time Series**					
Intercept	−0.526	0.21	59.25	−2.51	<0.05
Condition					
Count Backwards					
Neurofeedback	0.114	0.047	349.24	2.45	<0.05
Gender					
Female	0.17	0.072	50.23	2.38	<0.05
Male					
Medication Presence					
No Medication	0.223	0.093	102.06	2.40	<0.05
Medication Present					
Group by Linear Slope					
Controls × Linear Slope	−0.051	0.015	393.87	−3.50	<0.01
Depressed × Linear Slope	−0.001	0.012	395.90	−0.07	0.94
Parental Support	0.014	0.005	49.71	2.69	<0.01

**Table 3:** Whole-brain analysis results for neurofeedback versus count-backward conditions.

	Cluster Size (K)	Hemisphere	MNI Coordinates			*F*	*p*(K)
			x	y	z		
Main Effect of Neurofeedback							
Superior, Middle, and Inferior Temporal Gyrus, Insula BA 13, 21, 38, 47	1323	Left	−28	12	−18	137.12	<0.001
Left Cerebellum	602	Left	−20	−74	−36	108.83	<0.001
Fusiform, Parahippocampal, Middle, Inferior and Superior Temporal Gyrus, BA 13, 19, 21, 22, 36, 37, 39, 40	3925	Right	38	−86	20	108.13	<0.001
Posterior Cingulate Cortex, Precuneus, BA 23, 30, 31	546	Left	−06	−50	22	104.23	<0.001
Parahippocampal and Fusiform Gyrus, Left Cerebellum, BA 19, 36, 37	768	Left	−28	−34	−18	97.14	<0.001
Superior and Inferior Temporal Gyrus, Occipital Lobe, Inferior Parietal Lobule, Cuneus BA 19, 22, 27, 39, 40	2126	Left	−44	−80	10	96.35	<0.001
Superior Temporal, Middle and Inferior Frontal Gyrus, Insula, BA 47, 13, 45, 38, 46	1097	Right	36	26	00	97.66	<0.001
Right Cerebellum	477	Right	28	−74	−34	77.71	<0.001
Postcentral Gyrus, BA, 2, 40	504	Left	−46	−36	44	74.45	<0.001
Medial and Superior Frontal Gyrus, ACC, BA 9, 10, 11, 24, 32	1862	Left and Right	−06	56	14	73.38	<0.001
Precentral Gyrus, BA 6, 9	234	Right	38	−04	46	68.21	<0.001
Superior Frontal Gyrus, BA, 6, 8	362	Left and Right	02	18	56	63.95	<0.001
**Group by Condition Interaction**							
Superior and Middle Temporal Gyrus, BA 21, 22	163	Right	54	−48	08	17.67	<0.001

BA: Brodmann’s Area; H: Hemisphere.

## Data Availability

The data presented in this study are available at the request of the corresponding author. The data is not publicly available due to privacy or ethical restrictions.

## Supplementary Materials

The following supporting information can be downloaded at: https://www.mdpi.com/article/10.3390/biology12111399/s1, Figure S1: Acquisition Parameters; Table S1: Activity during Neurofeedback Minus Rest Associated with Main Effect of Neurofeedback; Figure S2: Brain Activity during Neurofeedback Minus Rest Associated with Main Effect of Neurofeedback. Figure S3: Mean ACC Activity across the Time-Series of the Neurofeedback Training. Table S2: Type III Tests for LMM Analysis of Mean AMYHIPPO Activity and Mean ACC Activity during NF Task. Table S3: Follow-Up T-Test for Whole-Brain Analysis. Table S4: Types of Medication (antidepressant) used by participants. References [77,114,115,116,117,118,119,120,121,122,123] are cited in Supplementary Materials.
