# Supplementary material for: Neurofeedback and Affect Regulation Circuitry in Depressed and Healthy Adolescents
Source: Biology (Basel). 2023 Nov 3;12(11):1399. doi: 10.3390/biology12111399 (PMC10669603; doi:10.3390/biology12111399)
Supplement: Supplementary file 1 [file biology-12-01399-s001.zip › biology-2574427-Supplementary Materials.pdf]

biology

MDPI

Supplements

1. Online Steps to Preprocess and Warp the ROI to Each Individual Brain

The single band reference functional image from a pre-feedback multiband EPI series was used as the target functional reference for the coordinate system transformation since the neurofeedback would be generated from subsequent real-time multiband EPI within the same imaging session. The high-resolution structural image of the subject (MPRAGE) was also used as a structural anatomic reference for registration to the MNI reference. The 4-step process was as follows: (1) Alignment of the subject's functional and structural images series. (2) Segmentation and spatial normalization of the structural image to the MNI coordinate space and output of spatial normalization parameters that perform inverse deformation between coordinate spaces. (3) Warping of the ROI from MNI space to subject space using the spatial normalization parameters obtained from step 2. (4) Registration of the warped ROI to match voxel-for-voxel to the specific subject's functional image space to allow for real-time masking. Masks were converted from 8 bit to 16 bit to be compatible with MURFI. The ROIs obtained from step 4 were then overlapped on both structural and functional images. See Supplementary Figure S1.

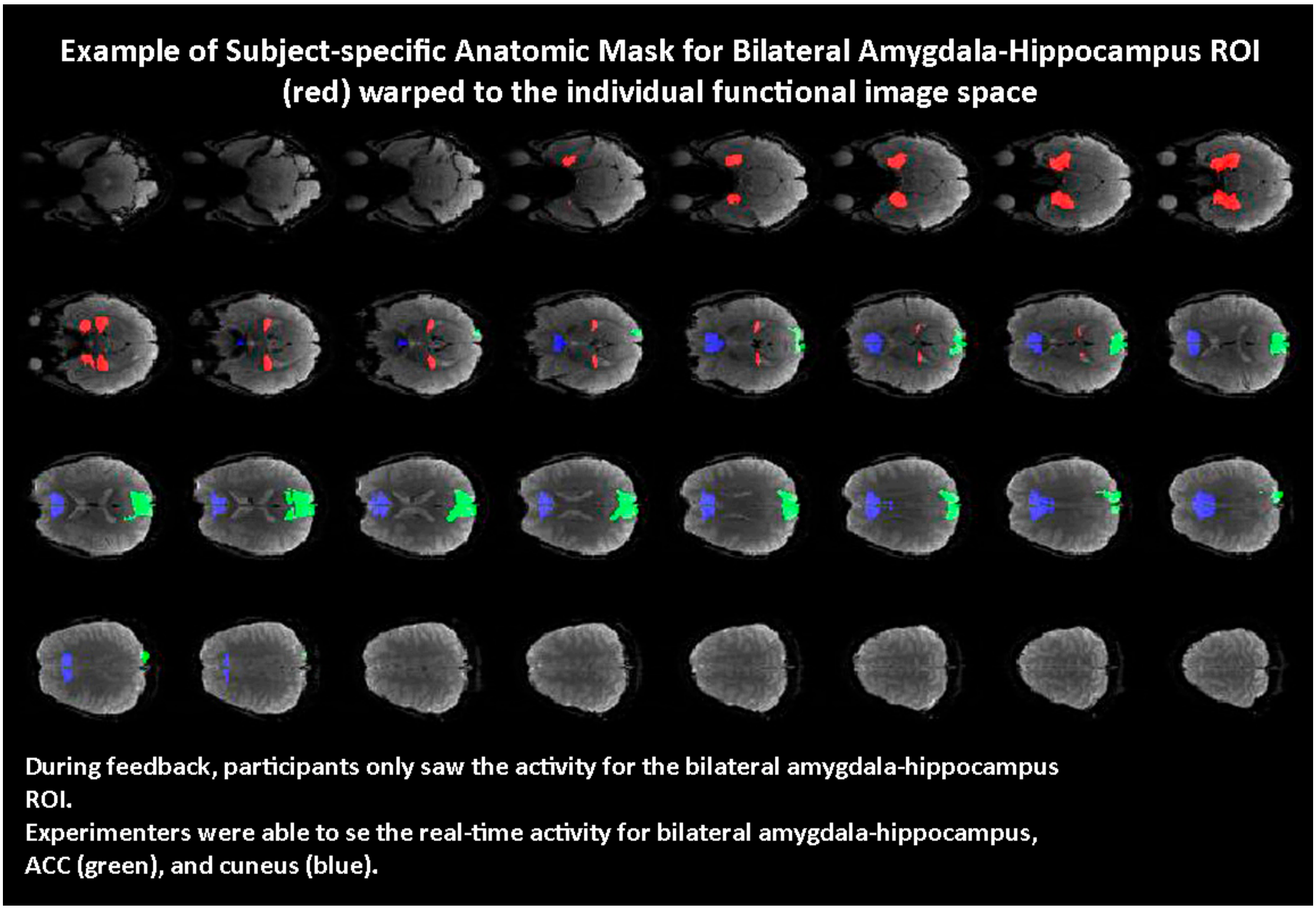

Figure S1. Acquisition Parameters. Neuroimaging data were collected using a 3.0 Tesla Siemens Prisma MRI scanner with the 32 channel receive-only head coil. Structural 3D axial MPRAGE images were acquired for each participant (TR/TE: 2100 ms/3.65 ms; TI: 1100; Flip Angle 7°; Field of View: 256 × 256 mm; Slice-Thickness: 1 mm; Matrix: 256 × 256; 224 continuous slices), GRAPPA 2. Mean (Blood Oxygenation Level Dependent) BOLD images were then acquired with a gradient echo EPI sequence during 6.08 minutes for the ESOM_Pre and Post tasks and 6.02 minutes for the ESOM-NF task, covering 60 oblique axial slices (2.4 mm thick; TR/TE = 1510/32.4 ms; FOV = 216 × 216 mm;

matrix 90 × 90; Flip Angle 65°) multi-band acceleration factor 3, resulting in a 2.4 × 2.4 × 2.4 mm 3 voxel size.

2. Offline Preprocessing and First Level Analyses

SPM12 was used for fMRI preprocessing and statistical analyses. Preprocessing the EPI time series included: (1) rigid body realignment for head motion correction, (2) slice timing correction, (3) rigid body co-registration of EPI with high resolution anatomical data, (4) spatial normalization to the Montreal Neurological Institute (MNI) anatomical space using unified segmentation, and (5) spatial smoothing (6 mm full width at half maximum). Head motion outliers in EPI time series were identified using the Artifact Detection Tools with a scan-to-scan movement threshold of 1 mm and a global scan-to-scan global signal change of 3 mm (www.nitrc.org/projects/artifact_detect/ (accessed on 20 January 2020).

For each subject, a BOLD-contrast signal variance was modeled with a set of regressors using a general linear model. The total signal variance was decomposed into a task component, with inter-trial intervals as implicit baselines. Each task regressor was constructed by generating condition duration vectors and then convolving them with a canonical hemodynamic response function, allowing parameter estimates proportional to task-related neural activity per second. The full model for each subject comprised: (1) the condition regressors, (2) regressors modeling movement-related signal modulation, (3) outlier time points, (4) the mean signal for the session, and (5) a discrete cosine transformation basis set that modeled the low frequency, presumably artifactual, signal modulations at frequencies lower than 0.008 Hz. Parameter estimates were calculated using restricted maximum likelihood algorithms.

3. Brain Areas during Neurofeedback Minus Rest

A full factorial GLM with NF task blocks as within subject's condition (FB vs. Rest) and IQ as covariate were ran. An effect of FB versus Rest condition showed that all participants had a higher bilateral superior, middle and inferior frontal gyrus, ACC, superior temporal gyrus, insula, left cerebellum, precentral, and right putamen, postcentral, and precuneus middle temporal gyrus during FB vs. Rest condition. However, higher activity was elicited during the Rest vs. FB in the right insula, superior temporal, postcentral, and precentral (supplement Table S1, Figure S2).

Table S1. Activity during Neurofeedback Minus Rest Associated with Main Effect of Neurofeedback.

Whole-Brain Results	p(K)	Cluster Size (K)	Hemisphere	MNI Coordinates			F
				X	y	z	
**Main Effect of Neurofeedback**							
Cerebellum	$p < 0.001$	875	Left	−30	−60	−28	133.28
Middle and Inferior Frontal, Superior Temporal Gyrus Precentral, Insula, Putamen, BA 6, 8, 9, 13, 44, 45, 46, 47	$p < 0.001$	3021	Right	32	26	00	223.56
Inferior Frontal, Superior Temporal Gyrus, Insula, BA 13, 45, 47	$p < 0.001$	898	Left	−30	24	00	117.19
Insula, Superior Temporal, Postcentral, Precentral, BA 6, 13, 22, 40, 41, 42, 43	$p < 0.001$	1081	Right	38	−16	18	108.66
Precentral, Middle and Inferior Frontal Gyrus, BA 6, 9, 46	$p < 0.001$	989	Left	−42	−04	46	101.50
Postcentral, Precuneus, Middle Temporal Gyrus, BA 2, 7, 40	$p < 0.001$	654	Right	32	−46	44	74.48

Superior and Middle Frontal Gyrus, Anterior Cingulate Cortex, BA 6, 8, 24, 32	$p < 0.001$	1715	Right and Left	−04	08	58	159.01

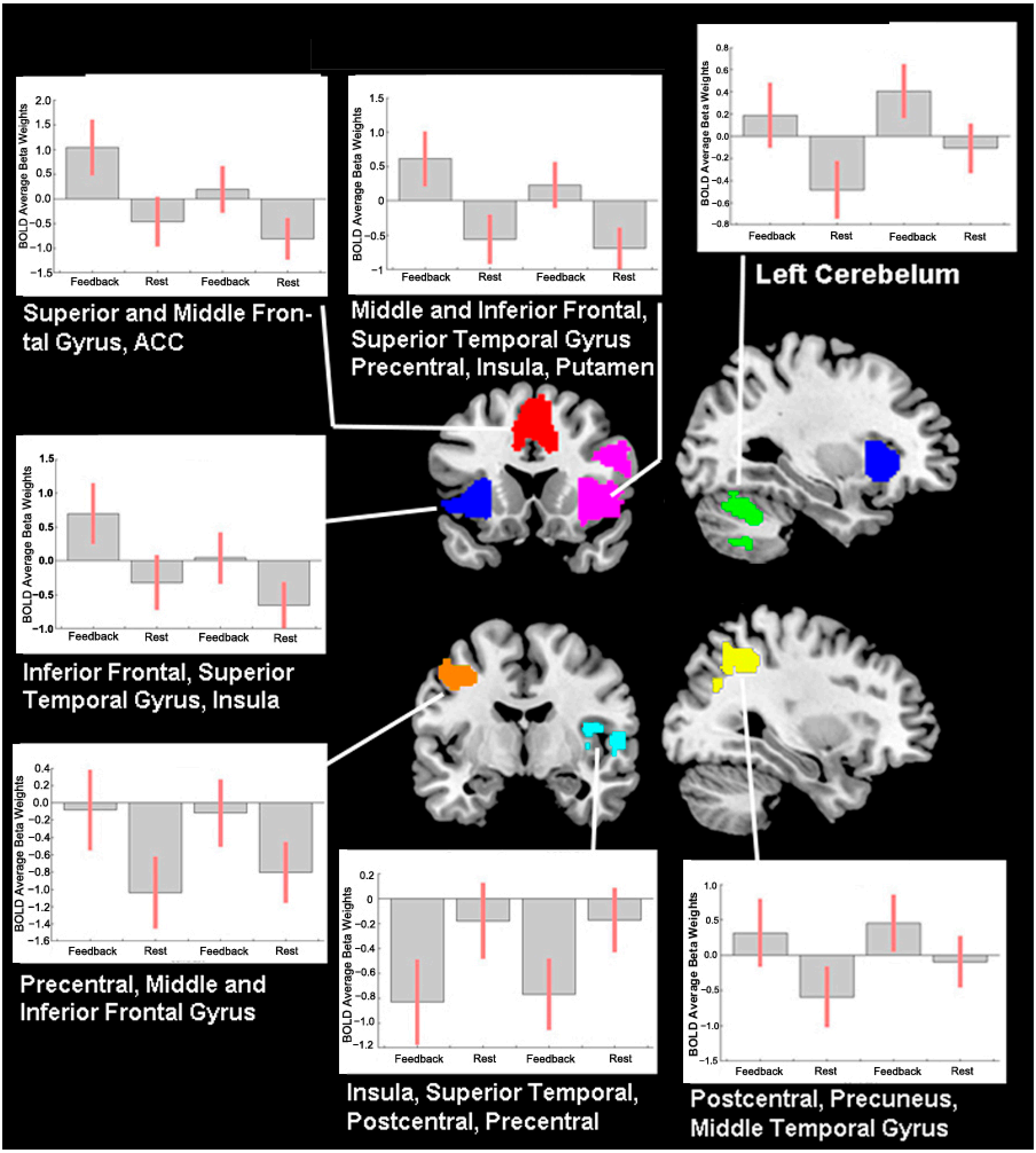

Figure S2. Brain Activity during Neurofeedback Minus Rest Associated with Main Effect of Neurofeedback. 90% confidence interval.

4. Linear Mixed Model (LMM) of AMYHIPPO and ACC Activity and Type III LMM Results

NF AMYHIPPO Initial Model with 17 Predictors of Interest: Linear, Quadratic, Cubic, Task_Condition (FB or CB) Diagnostic_Group (Depressed or Control), Gender, Medication Presence, Parental Support, IQ, Self Esteem, Suicide Ideation, Depression, Behavioral Inhibition vs. Approach, Conflict with parents, Self-Injury, Rumination Change, Depression Change. Models were compared via a χ^2 test of the −2 LL fit difference. The more complex model after removing non-significant variables had 4 predictors, whereas the simpler model had 3 predictors and compared to the larger model the −2 LL test yielded: $-2\ LL_{smaller\ model} - 2\ LL_{larger\ model} = 1.74$, df = 1 (NS), compared to the critical $\chi^2_{(1)} = 3.84$. Therefore, the simpler model fitted the data better.

NF ACC Initial Model with 17 Predictors of Interest: Linear, Quadratic, Cubic, Task_Condition (FB or CB) Diagnostic_Group (Depressed or Control), Gender, Medication Presence, Parental Support, IQ, Self Esteem, Suicide Ideation, Depression, Behavioral Inhibition vs. Approach, Conflict with parents, Self-Injury, Rumination Change, Depression Change. Models were compared via a χ^2 test of the −2 LL fit difference. The more complex model after removing non-significant variables had 5 predictors, whereas the simpler model had 4 predictors and compared to the larger model the −2 LL test yielded: $-2\ LL_{smaller\ model} - 2\ LL_{larger\ model} = 6.699$, df = 1 (NS), compared to the critical $\chi^2_{(1)} = 3.84$. Therefore, the fuller model fitted the data better.

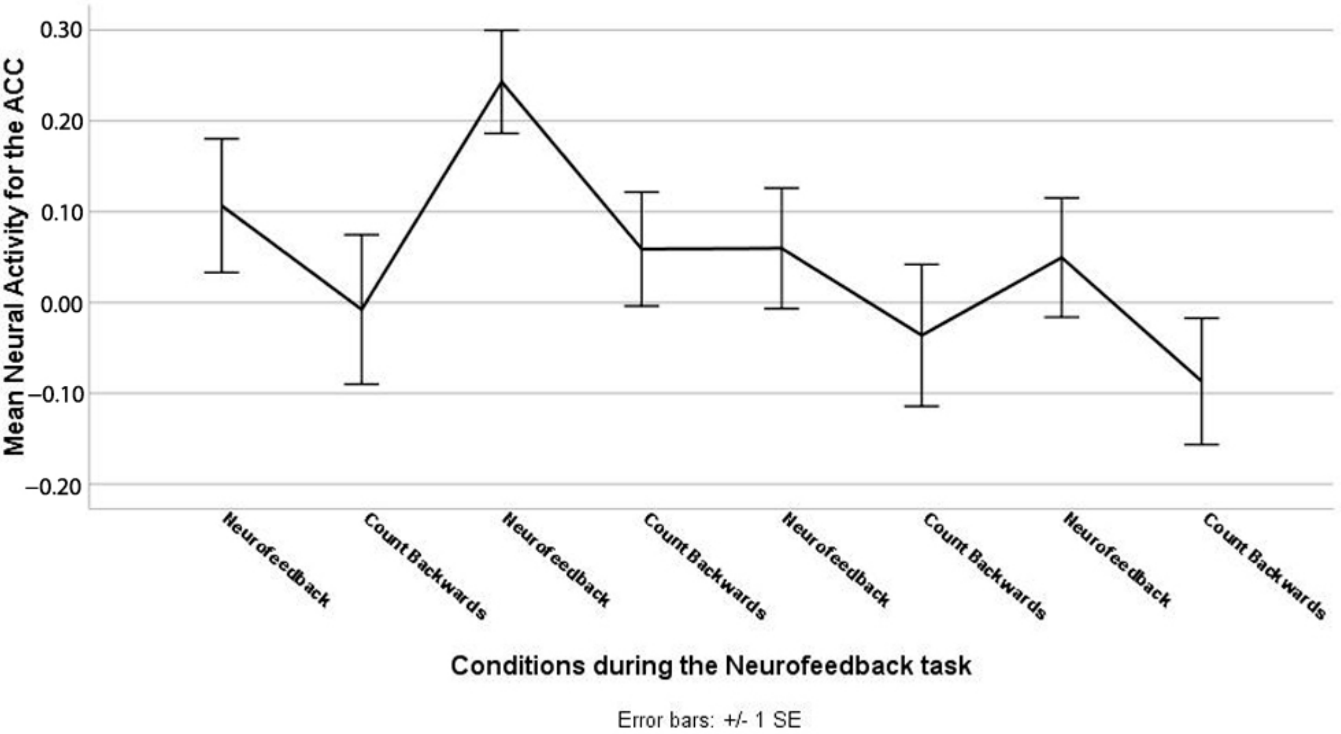

Figure S3. Mean ACC Activity across the Time-Series of the Neurofeedback training.

5. LMM Analysis of Mean AMYHIPPO or ACC Activity Significant Predictors

Table S2. Type III Tests for LMM Analysis of Mean AMYHIPPO Activity and Mean ACC Activity During NF Task

AMYHIPPO				
**Effect**	**Numerator df**	**Denominator df**	***F***	**Sig. p**
Intercept	1	211.02	8.40	<0.01
Group	1	203.96	12.26	<0.01
Condition	1	364	8.79	<0.01

Group by Linear Slope	2	364	8.26	<0.01
**ACC**				
Intercept	1	55.26	3.74	0.058
Condition	1	349.24	6.02	<0.05
Gender	1	50.23	5.65	<0.05
Medication Presence	1	102.06	5.75	<0.05
Group by Linear Slope	2	366.06	6.35	<0.01
Parental Support	1	49.71	7.21	<0.01

6. Table S3. Follow-Up T-Test for Whole-Brain Analysis

Whole-Brain Results	p(K)	Cluster Size (K)	Hemisphere	MNI Coordinates			T
				X	y	z	
**Neurofeedback (NF) > Count Backwards (CB)**							
Superior, Middle, and Inferior Temporal Gyrus, Insula BA 13, 21, 38, 47	$p < 0.001$	1492	Left	−28	12	−18	11.71
Left Cerebellum	$p < 0.001$	662	Left	−20	−74	−36	10.43
Fusiform, Parahipocampal, Middle, Inferior and Superior Temporal Gyrus, BA 13, 19, 21, 22, 36, 37, 39, 40	$p < 0.001$	4191	Right	38	−86	20	10.40
Posterior Cingulate Cortex, Precuneus, BA 23, 30, 31	$p < 0.001$	585	Left	−06	−50	22	10.21
Parahippocampal and Fusiform Gyrus, Left Cerebellum, BA 19, 36, 37	$p < 0.001$	3207	Left	−28	−34	−18	9.86
Superior and Inferior Temporal Gyrus, Occipital Lobe, Inferior Parietal Lobule, Cuneus BA 19, 22, 27, 39, 40	$p < 0.001$	3207	Left	−44	−80	10	9.86
Superior Temporal, Middle and Inferior Frontal Gyrus, Insula, BA 47, 13, 45, 38, 46	$p < 0.001$	1242	Right	36	26	00	9.88
Right Cerebellum	$p < 0.001$	518	Right	28	−74	−34	8.82
Postcentral Gyrus, BA, 2, 40	$p < 0.001$	3207	Left	−46	−36	44	9.86
Medial and Superior Frontal Gyrus, ACC, BA 9, 10, 11, 24, 32	$p < 0.001$	2167	Left and Right	−06	56	14	8.57
Precentral Gyrus, BA 6, 9	$p < 0.001$	276	Right	38	−04	46	8.26
**Con NF > Con CB = Dep NF = Dep CB**							
Superior and Middle Temporal Gyrus, BA 21, 22	$p < 0.001$	333	Right	54	−48	08	3.81

BA = Brodmann's Area. Con = Healthy Controls. Dep = Adolescents with Depression.

7. Ratings for Happiness and Memory Recall

A 10-point scale rating measured successful recalling of happy memories during the NF task and happiness before and after NF. There were no significant differences, $F(1,48) = 0.397$, $p = 0.53$, for ratings in successful recalling happy memories between control (M = 5.53) and depressed (M = 5.18) groups. Additionally, analyses of ratings for happiness before and after the NF task showed that healthy control youth tended to have a higher rating in happiness overall at both times. However, the differences between group were not significant, $F(1,50) = 2.91$, $p = 0.09$, and the groups did not differ in their ratings before and after the NF task $F(1,50) = 2.91$, $p = 0.09$.

8. Table S4. Types of medication (antidepressant) used by participants

Types of Medication	Number of Participants

Fluxetine	7
Lexapro	5
Wellbutrin	5
Prozac	4
Celexa	3
Sertraline	2
Velafaxine	2
Bupropion_Wellbutrin	1
Citalopram	1
Cymbalta	1
Fluoretine	1
Lamictal	1
Abilify	1
Aripiprazole	1
Trazodone	1

9. Recruitment and Inclusion Criteria

Participants were recruited from the community and from inpatient units at the University of Minnesota. Exclusion criteria included the following: General MRI exclusions, psychosis, major medical or neurological disorders and meeting criteria for substance abuse or dependence. During the first session, participants completed questionnaires for parental support via the Emotional Socialization Measure [ESM, [77]], IQ was sampled [WASI, [114]]. Participants completed questionnaires measuring, suicide ideation via the About My Life questionnaire[115], self-esteem via the perceived competence scale for children [PCSC, [116]], depression [BDI, [117]], behavioral inhibition vs. approach [BISBAS, [118]], conflicts with parents [CBQ, [119]], self-injury [Deliberate Self-Harm Inventory, [120]], rumination [121] and a depression questionnaire [122,123] during the intake first session. In addition to the first session, rumination and depression were also sampled before and after scanning during the second session. Future research will examine whole brain correlates of change in these measures.

References [77,114-123] are cited in the main texts.
